# Advancements in biohydrogen production – a comprehensive review of technologies, lifecycle analysis, and future scope

**DOI:** 10.1039/d4ra06214k

**Published:** 2024-11-18

**Authors:** Aarnav Hetan Sanghvi, Amarjith Manjoo, Prachi Rajput, Navya Mahajan, Natarajan Rajamohan, Iyman Abrar

**Affiliations:** a Department of Electrical & Electronics Engineering, Birla Institute of Technology and Science, Pilani – Hyderabad Campus Shameerpet Hyderabad Telangana-500078 India; b Department of Chemical Engineering, Birla Institute of Technology and Science, Pilani – Hyderabad Campus Shameerpet Hyderabad Telangana-500078 India iyman@hyderabad.bits-pilani.ac.in; c Chemical Engineering Section, Faculty of Engineering, Sohar University Sohar P C-311 Oman rnatarajan@su.edu.om

## Abstract

The global shift towards sustainable energy sources, necessitated by climate change concerns, has led to a critical review of biohydrogen production (BHP) processes and their potential as a solution to environmental challenges. This review evaluates the efficiency of various reactors used in BHP, focusing on operational parameters such as substrate type, pH, temperature, hydraulic retention time (HRT), and organic loading rate (OLR). The highest yield reported in batch, continuous, and membrane reactors was in the range of 29–40 L H_2_/L per day at an OLR of 22–120 g/L per day, HRT of 2–3 h and acidic range of 4–6, with the temperature maintained at 37 °C. The highest yield achieved was 208.3 L H_2_/L per day when sugar beet molasses was used as a substrate with *Clostridium* at an OLR of 850 g COD/L per day, pH of 4.4, and at 8 h HRT. The integration of artificial intelligence (AI) tools, such as artificial neural networks and support vector machines has emerged as a novel approach for optimizing reactor performance and predicting outcomes. These AI models help in identifying key operational parameters and their optimal ranges, thus enhancing the efficiency and reliability of BHP processes. The review also draws attention to the importance of life cycle and techno-economic analyses in assessing the environmental impact and economic viability of BHP, addressing potential challenges like high operating costs and energy demands during scale-up. Future research should focus on developing more efficient and cost-effective BHP systems, integrating advanced AI techniques for real-time optimization, and conducting comprehensive LCA and TEA to ensure sustainable and economically viable biohydrogen production. By addressing these areas, BHP can become a key component of the transition to sustainable energy sources, contributing to the reduction of greenhouse gas emissions and the mitigation of environmental impacts associated with fossil fuel use.

## Abstract 2

The global shift towards sustainable energy sources, necessitated by climate change concerns, has led to a critical review of biohydrogen production (BHP) processes and their potential as a solution to environmental challenges.

## Graphical abstract

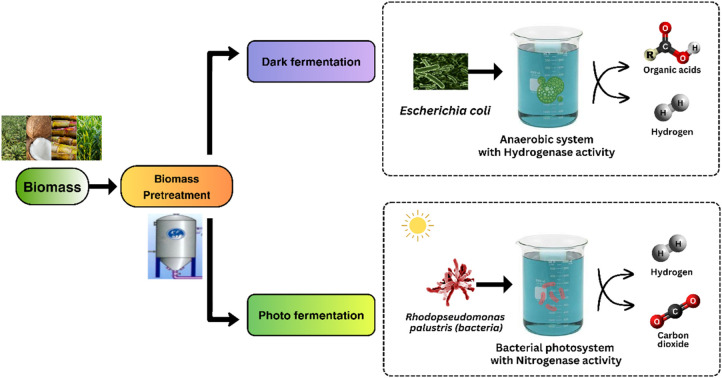

## 1. Introduction

Demand for fossil fuels has increased multifold, creating an imbalance between production and consumption. It has become increasingly important to rely on environmentally clean fuels like hydrogen. Hydrogen, a very efficient fuel with one of the highest calorific values (120–142 MJ kg^−1^), is considered as a clean fuel because it releases only water vapour as a byproduct during combustion.^1^ Out of the several methods available to generate hydrogen, biological processes have long been preferred due to favourable operating parameters like low temperature and pressure and low costs of operation.^2^ Among the different renewable energy resources available, waste biomass has proven to be one of the most potent choices for producing green fuels.^3^ Biohydrogen Production (BHP) is a propitious domain for sustainable energy generation, with its viability depending on substrate costs and pretreatment technologies. Fermentation of carbohydrates from organic substrates like agricultural biomass holds potential, yet their complex structure poses efficiency challenges.^4^

Two primary BHP technologies utilizing biomass as fuel are thermochemical and biological processes. Dark fermentation, carried out by fermentative bacteria, utilizes a variety of organic biomasses to produce biohydrogen and organic acids.^5^ Photo fermentation, which involves photosynthetic purple non-sulfur (PNS) bacteria, produces biohydrogen from organic acids under anaerobic, light-driven conditions.^6^ Biohydrogen production offers significant environmental and operational benefits over conventional chemical hydrogen generation methods. Traditional chemical processes, like steam methane reforming and coal gasification, are energy-intensive and heavily reliant on fossil fuels, resulting in high greenhouse gas emissions.^7^ In contrast, BHP uses renewable feedstocks, such as agricultural waste and wastewater, creating a sustainable, low-emission hydrogen source. BHP systems typically operate under milder conditions, which not only reduces energy costs but also enables simpler process setups.^8^ Understanding the kinetics of these processes and optimizing conditions through pretreatment methods are crucial for enhancing efficiency and economic viability in BHP.

Different types of reactors play an important role in BHP and their design and affecting parameters influences the yield of production. The batch reactor provides BHP with advantages such as flexibility, robust control strategy implementation, and reduced cost. However, challenges exist, including the need for substrate pretreatment, low hydrogen yields from complex organic materials, and the difficulty in achieving high hydrogen yields due to biochemical and thermodynamic constraints.^9^ Research efforts to enhance the efficiency of BHP processes include modelling, optimization, metabolic engineering, using mixed cultures, and changing reactor layouts.^10^ Furthermore, the comparison between continuous and batch reactors indicates that batch reactors may yield higher hydrogen rates due to varying substrate concentrations but only in the early stages of the batch, else continuous reactors are more efficient.^11^ Continuous stirred tank reactors (CSTRs), packed bed reactors (PBRs) and membrane bioreactors (MBRs) play a vital role in the continuous production of biohydrogen. A short hydraulic retention time (HRT) and washout of biomass have been identified as constraints in CSTR performance.^12^ Additionally, the integration of CSTRs with other bioreactors, such as upflow anaerobic sludge blanket (UASB) reactors, has been explored for simultaneous hydrogen and methane production. PBRs are known for their simple design with minimal moving parts, which makes them cost-effective and easy to operate. PBRs offer a wide range of operational times and high biomass concentrations, as the microorganisms attach to the solid support material, leading to long operational periods.^13^ A highest BHP rate was achieved at 2 h HRT for PBRs operated at various HRTs varying from 1.2 to 24 h, and using paper mill effluent as substrate.^14^ However, PBRs struggle with clogging from complex substrates, uneven flow distribution, and optimal temperature maintenance. MBRs have emerged as a more efficient BHP alternative, when particularly combined with biological processes. MBRs show higher hydrogen yields when compared to PBRs^15^ and they also provide better control over microbial diversity and process stability, leading to more consistent BHP rates.^16^ The addition of biofilm support was observed to result in BHP rate of 44.22 L/L per day, when polyester screen mesh was used compared to BHP rate of 51.64 L/L per day with stainless-steel.^17^ MBRs, though they offer higher yields and better control, they come with their own drawbacks such as membrane fouling. Both PBRs and MBRs have limitations in their microbial communities, requiring further research to understand their full potential.

In recent years, several machine learning (ML) models have been implemented to boost the efficiency of BHP. Artificial intelligence (AI) tools help in predicting and alleviating problems that might be faced during the process. Some important and frequently-used AI tools such as artificial neural network (ANN) models used to estimate BHP yield and support vector machines (SVM) models used to classify errors have been discussed in the paper. By integrating AI tools such as ANNs and SVMs into the biohydrogen production process, it is possible to achieve significant improvements in efficiency, reliability, and yield. These tools enable precise predictive modeling, process optimization, anomaly detection, and automated control, ultimately leading to a more sustainable and economically viable BHP system. Before commercializing biohydrogen produced through any methods, doing a thorough life cycle assessment (LCA) and technoeconomic analyses (TEA) is very important. TEA gives an idea about the financial viability of a biological procedure by examining economic aspects involved during the production process.^16,17^ LCA analyses the effect the procedure would have on the environment throughout the life cycle.

The existing literature on BHP reveals critical gaps, particularly in reactor design and the integration of AI-driven optimization methods. The low yields of dark and photofermentation processes demand advanced reactor designs, often complex and resource-intensive. While scaling up reactor systems for diverse feedstocks has potential to enhance efficiency, it remains a formidable challenge. AI models, including ANN and Adaptive Neuro-Fuzzy Inference Systems (ANFIS), are underutilized in this field despite their proven effectiveness in other biotechnological applications for process parameter optimization and yield prediction. The lack of real-time AI-driven monitoring and control also restricts the ability to dynamically adjust reactor conditions, thereby limiting efficiency, adaptability, and scalability improvements across various operating conditions.^18^ Another notable gap includes sustainable management of post-fermentation broth, which is critical for reducing environmental impact but often overlooked in biohydrogen processes. Current strategies lack comprehensive waste management approaches that incorporate reuse, recycling, or safe disposal practices, which are essential for a sustainable BHP lifecycle. Addressing these gaps with integrated waste management solutions could facilitate the broader environmental acceptance and practical applicability of biohydrogen.^19^

This review offers an in-depth analysis of light and dark fermentation, exploring their operational mechanisms and contextual applications, and provides insights into biohydrogen yield optimization for various reactor types, while detailing their respective advantages and constraints. Furthermore, it introduces AI-driven prediction tools to predict reactor performance represents a novel approach to enhance process optimization and gain deeper insights into the dynamic behaviour of BHP processes. The review also considers the broader sustainability and economic implications of BHP, with comprehensive assessments *via* LCA and TEA to align with global sustainability and energy transition objectives. Future research should prioritize integrated cultivation and waste management systems to address both efficiency and environmental impact. Expanding the role of real-time AI-based monitoring and process control in BHP, alongside adaptable reactor designs, could enhance operational precision and pave the way for sustainable scale-up and industrial application.

## 2. Technology overview and mechanism

**Fig. 1:**
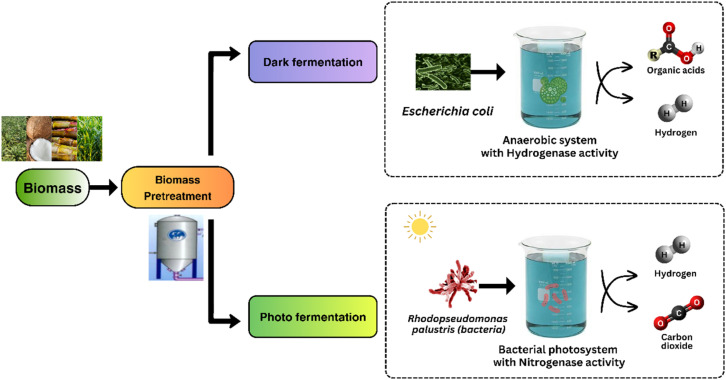
Different pathways of dark and photo fermentation for BHP.

The technological and kinetic viability of BHP is majorly influenced by the costs associated with the substrate and also the technology used for pretreatment process. In accordance with fermentation principles, the conversion of biohydrogen from carbohydrates, particularly those found in organic substrates like agricultural biomass, holds considerable promise. Nevertheless, the intricate and heterogeneous structure of matrices poses challenges to the efficient fermentation of carbohydrates.^20^ To overcome this obstacle, substrates necessitate pretreatment to liberate more accessible carbohydrates which eventually enhances fermentation efficiency.^21^ Selecting the most suitable pretreatment method depends on the substrate type and composition of the substrate.^22^ The pre-existing pretreatment methods, pyrolysis and ultrasound performs better in the case of cell destruction and needs lesser treatment time. Although, these techniques require high energy conditions which can lead to further breakdown of sugars, hence becoming unsuitable.^23^ While pyrolysis, combustion, liquefaction and gasification are key thermo-chemical processes that use biomass for hydrogen generation, direct and indirect bio-photolysis, dark, photo, and integrated fermentation (a combination of both dark and photo fermentation) are the main microbial processes.^4^ The different pathways of dark and photo fermentation for BHP has been depicted in Fig. 1.

### 2.1. Dark fermentation for BHP

Several fermentative bacteria (either obligatory or facultative) produce biohydrogen through dark fermentation and may use a larger variety of organic biomass or wastes as substrate. The capacity of dark-fermentative bacteria to degrade anaerobic or anaerobic biomass distinguishes them as obligatory or facultative anaerobes. The fermentative bacteria convert organic substrates into hydrogen and other by-products under anaerobic conditions. This process is carried out in the absence of light, distinguishing it from photofermentation. The primary reactions involved in dark fermentation include the breakdown of carbohydrates into simpler compounds, resulting in the production of H_2_, CO_2_, and volatile fatty acids (VFAs) such as acetic acid and butyric acid.^24^ The accumulation of these acids can affect the overall yield and efficiency of hydrogen production, making their monitoring and control essential for optimizing the process. Dark fermentation commonly produces hydrogen yields of 1–2 moles per mole of carbohydrates consumed, depending on the microbial strain and operating conditions. The process efficiency is influenced optimal pH, correct temperature and the extent of byproducts produced. Many studies report optimal conditions at a pH of 5.5–6.0 and temperatures around 35–37 °C for hydrogen-producing *Clostridium* strains, maximizing yields close to 2.0 moles H_2_ per mole of glucose. Organic acids like acetate and butyrate are byproducts, with yields of up to 2–3 g L^−1^ depending on the carbon source and fermentation conditions.^25^

The kinetic parameters of biohydrogen generation, which rely on the kind of substrates being fermented and the inoculum microbial population, characterize the activities of the dark fermentation process and span a large range. The formation of dark fermentative biohydrogen has been extensively described using kinetic models, such as the Modified Gompertz Model given by eqn (1) which discusses the regular changes in the growth rate of biohydrogen producing bacteria substrate concentrations, and the Modified Logistic Model given by eqn (2) which aid in defining the precise functions of factors that affect H_2_ yield and aid in process design.^5^1
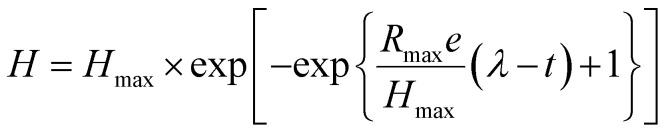
2
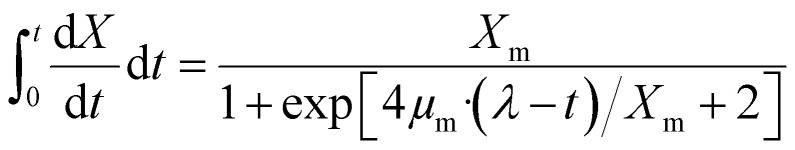
where in eqn (1), *H* and *H*_max_ tell the cumulative degraded substrate value and the maximum degraded substrate value, used to describe the degradation progress. In eqn (2), *X* (g L^−1^) is the biomass at the reaction time *t*, *X*_m_ (g L^−1^) is the maximum biomass, *λ* (h) is the lag time and *μ*_m_ (g/L per h) is the maximum growth rate.

In dark fermentation, microbial contamination impacts hydrogen production by introducing non-hydrogen-producing organisms that consume essential substrates or even the hydrogen itself, reducing yield. Contaminants like methanogens can outcompete hydrogen-producing bacteria such as *Clostridium* and *Enterobacter* by using substrates like acetate and butyrate, diverting the process toward methane production instead of hydrogen. To address this, pretreatment techniques—such as heat, chemical, or pH treatments—help deactivate unwanted microbes, creating an environment where hydrogen producers can thrive. Additionally, metabolic engineering of hydrogen-producing microbes is explored to improve their substrate utilization and resistance to contamination, potentially increasing hydrogen output.^26^

### 2.2. Photo fermentation for BHP

Under anoxygenic conditions using light as their energy source, photosynthetic PNS bacteria may produce hydrogen from organic acids (butyrate, acetate, succinate, malate, *etc.*) and CO_2_.^27^ Consequently, photo-fermentation offers the possibility of generating hydrogen from a variety of substrates, such as wastewater and organic acid-rich waste.^28^ Depending on the carbon source, the literature indicates a maximum light conversion efficiency of 9.3% and a maximum hydrogen production of 80%. The chemical oxygen demand (COD) removal efficiency and hydrogen output of photo-fermentation are both high in theory, but their economic viability is constrained by the activity of the hydrogen-producing enzyme (nitrogenase) and light intensity.^29^ Several PNS bacteria strains, including *Rhodobacter sulfidophilus*, *Rhodopseudomonas palustris*, *Rhodobacter sphaeroides O.U001*, *Rhodobacter capsulitis*, *R. sphaeroides RV*, and others, have been examined for photo fermentative hydrogen generation. In this fermentation, photosynthetic bacteria like *Rhodobacter* species can achieve theoretical yields of up to 3.5–4 moles H_2_ per mole of organic acid under optimal conditions, though practical yields often range around 2.5–3.0 moles. Light intensity plays a key role; low light can limit yield, while high light can induce photoinhibition. Typical light intensities of 4000–6000 lux are reported to support optimal conversion rates, though reactor design and light penetration are significant limiting factors.^30^Eqn (3) illustrates the hydrogen production from acetate *via* photo fermentative method.

3 2CH_3_COOH + 2H_2_O → 4H_2_ + 2CO_2_, Δ*G*_0_ = +104 kJ

The logistic equation for the fermentation process explains and discusses the growth kinetics of *Rhodobacter sphaeroides O.U001*. The logistic model, a sigmoidal-shaped model, has recently become the most popular due to its ‘goodness of fit’ and has been frequently utilized to describe the proliferation of microorganisms. The logistic model has the advantage of portraying the whole growth curve, including the lag phase (if present), exponential growth, and stationary periods. Eqn (4) is based on logistic model which accounts for growth-associated hydrogen generation in photo fermentation.^6^4
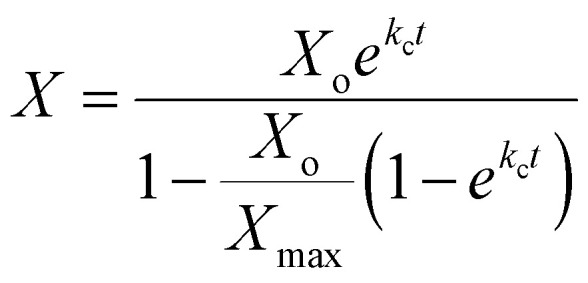


Eqn (4) depicts the corelation between biomass production concentration (*X*) and fermentation time (*t*) where *X* max and *k*, kinetic parameters are calculated using logistic curve.

Microbial contamination poses a challenge in photo-fermentation for hydrogen production as unwanted microbial growth competes for nutrients, reducing hydrogen yield. Contaminants can also disrupt the delicate balance of environmental conditions essential for photo-fermentative bacteria, such as *Rhodobacter* and *Rhodopseudomonas* species, thereby lowering efficiency. To address contamination, pre-treatment methods like pasteurization and pH adjustments are used to inhibit unwanted microbes without affecting target bacteria. Combining photo-fermentation with dark fermentation (two-stage fermentation) is another strategy that boosts yields and stabilizes pH, minimizing contamination issues.^31^

## 3. Reactor types

**Fig. 2:**
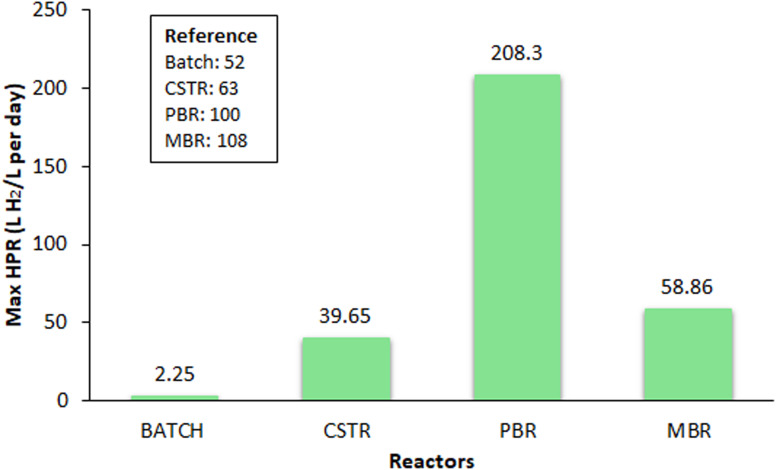
Comparison of reactor performance in terms of BHP rate.

The core of BHP technology depends upon the efficiency of bioreactors, specially engineered to optimize the microbial BHP processes. This section provides an in-depth exploration of different reactor configurations used in BHP, including batch reactor, CSTR, PBR, and MBR, with the maximum BHP rate represented in Fig. 2. Furthermore, a comprehensive understanding of the various parameters and several challenges encountered in the pursuit of efficient BHP have also been discussed. Parameters such as pH, temperature, substrate concentration, HRT must be optimized to maximize BHP while minimizing the operational costs and environmental impacts, and also taking other challenges such as system integration, and development of cost-effective reactor materials into consideration.

### 3.1. Application of batch reactor for BHP

Studies have reported that batch operation generally yields higher amounts of hydrogen compared to continuous operation, because bacteria in a batch reactor experience a distinct environment with varying substrate concentrations during the early stages of the batch compared to low-substrate microenvironment in continuous operation.^32^ A viable substitute for BHP using organic solid waste is the sequential batch reactor (SBR), which offers the advantages of flexible operation, comprehensive instrumentation possibilities, robust control strategy implementation, and reduced cost.^11^ An efficient feedstock-retaining reactor that runs on self-immobilized biomass is called an anaerobic sequencing batch reactor (ASBR). Therefore, if enhanced hydrogen-generating bacterial populations are attained, operational systems that uses ASBR have a higher chance of producing hydrogen for a longer period and at a higher yield. In addition to these benefits, the ASBR is superior to other systems because of its versatility, ease of use, and ability to settle within the same reacting vessel.^33^

#### 3.1.1. Influence of operating variables

The performance of a reactor not only depends upon its design, but it is also highly influenced by a multitude of other parameters. These parameters range from the physical and chemical properties of the substrate to the operational conditions within the reactor, as given in Table 1. Reactors that are frequently run in fed-batch mode may produce high-value products at sluggish rates or with a poor yield.^47^ Batch system models are frequently undeviating in the feed rate, which is a governing parameter and nonlinear in the state variables. Determining the best control profile is crucial for optimizing product yield because the end product has a high value.^48^ When applied to SBR systems, a broad range of HRT varying from 6 to 72 h and solid retention time (SRT) varying from 16 to 264 h have produced varying hydrogen generation rates in order of 0.75–6.7 L of H_2_/L per day.^49^ BHP from *spirogyra* biomass was conducted in a SBR with a biogas collection and storage system, where the biomass was pre-treated with two acid hydrolysis before comparing the subsequent fermentation by *Clostridium Butyricum*. In order to facilitate the later characterization and storage of the biogas produced, the fermentation was gradually increased up to a batch-regulated bioreactor connected with a collecting system.^50^ Another research was carried out over a 24 h period in an ASBR operating at room temperature in an acidophilic microenvironment with a pH of 6, with the main goal being to assess the viability of using a bioaugmentation technique to improve BHP during the treatment of chemical wastewater. The research showed maximum hydrogen yield of 4.2 × 10^−5^ mol min^−1^ after 2 h of cycle operation, at an OLR of 5.6 g COD/L per day after the treatment of chemical wastewater.^51^

**Table 1:** Application of batch reactors for BHP and their operating conditions

Substrate	Micro-organism	OLR	pH	Temperature	HRT	BHP rate	Reference
Vinicultural biomass (grape residues)	*Clostridia* species	14 to 28 g COD/L	5.7 ± 0.5	37 ± 1 °C	24 h	2.2 × 10^−4^ mol H_2_ per min	34
Winery wastewater	*Lactobacillus*, *Pectinatus*, and *Clostridium* species	108.6 g COD/L per day	5.0–5.6	37 ± 1 °C	5.5 h	0.05 L H_2_/L per day	35
Palm oil mill discharge	Clostridiaceae	45.4 g COD/L per day	4.5 ± 2	60 ± 1 °C	8 h	6.1 ± 0.03 L H_2_/L per day	36
Tequila vinasses	*Clostridium* and *Bacillus* species	20 g COD/L per day	7.5	54.7 °C	29 h	0.15 L H_2_/L per h	37
Cheese whey	*Rhodobacter sphaeroides*	70.4 kg COD/m^3^ per day	4.25	28 ± 2 °C	8 h	3.78 ± 3.33 × 10^−3^ mol H_2_ per day	38
Food waste	*Enterococcus* and *Veillonella*	22 g COD/L per day	5.5–7.0	37 °C	3 days	0.73 ± 0.28 L H_2_/L per day	39
Apple pulp waste	*Sporolactobacillus* (39%), *Clostridium* (15%), and *Coprothermobacter* (10%)	100 g COD/L per day	7.2	37 ± 1 °C	12 h	0.07 L per g volatile solids	40
Cacao pod husk	*Ethanoligenes*	35 g COD/L per day	4.0–4.5	25.24–26.16 °C	78–103 h	0.25 ± 0.007 L/L per day	41
Potato waste	*Enterobacter aerogenes* MTCC2822	20 g COD/L per day	7.0	50 °C	48 h	1.58 L H_2_/L per day	42
Brewery wastewater	*Clostridia* species	38.44 g COD/L per day	4.13–4.72	55 °C	20 days	0.60 to 1.50 g/L per day	43
Cassava wastewater	*Eubacterium*	14 g COD/L per day	6.0	30 ± 1 °C	4 h	0.7 L H_2_/L per day	44
Citrus peel waste	*Escherichia* and *Clostridium*	30 g COD/L per day	5.5–8.5	30–44 °C	20.9 h	0.84 L H_2_/L per day	45
Crude glycerol	*Clostridiales*	20 g COD/L per day	5.5	37 °C	28 h	3.94 L H_2_/L per day	46

One of the most efficient ways for BHP in a batch reactor is using starch because of its of abundance, fermentability by various bacteria, high hydrogen yield, and cost-effectiveness. The hydrogen yield is capable of achieving 0.199–0.240 L_H_2__ g^−1^ in a batch reactor.^52^ It was found that the initial concentration given to the reactor had an impact on the amount of biogas and hydrogen generated, and the temperature and HRT had an impact on the extent to which this effect was produced.^10^ Using an ASBR without pH control, the batch biohydrogen yield of rice wastes (rice bran and rice straw) was assessed by combined assimilation with heat-shocked slurry under ambient and high temperatures. Due to its higher lignin concentration of 19.34% compared to rice straw, rice husk exhibited the lowest experimental yield under ambient conditions.^53^ The process of dark fermentation of palm oil mill discharge involves the evaluation of the two-stage process by employing enriched mixed culture in an ASBR system. It was observed that the two-stage ASBR system with thermophilic and mesophilic BHP was effective for both total suspended solids degradation and combined sugar utilization in palm oil mill discharge at the ideal HRT of 12 h.^54^

The impact of several parameters, including pH and temperature, on hydrogen output was assessed for BHP from food waste through dark fermentation. It was observed that at 5.5 pH, temperature of 37 °C, and 0.80 L of working volume, the hydrogen yield was 0.064 L g^−1^ the energy yield was 0.7 kJ g^−1^. Under other conditions, involving a 0.15 L working volume, 37 °C temperature, and pH of 5.5, the energy yield was 1.2 kJ g^−1^ and BHP was 0.106 L g^−1^. These results illustrate the manner in which operating conditions impact energy recovery and hydrogen-generating yield.^55^ In utilizing chemical wastewater as a substrate, a sequencing batch reactor configured with a biofilm was employed to generate hydrogen *via* fermentative processes. The results revealed a discernible correlation between the variation in OLR and the corresponding fluctuation in the rate of hydrogen production, indicating a decrease in hydrogen generation with higher OLRs.^51^

#### 3.1.2. Optimization strategies

Optimization of BHP in batch reactors involves a range of strategic adjustments and advanced modeling techniques. Key optimization strategies include altering the OLR, maintaining suitable effluent pH levels, and achieving optimal concentrations of mixed liquor suspended and volatile suspended solids, each of which can enhance biohydrogen yield significantly. Studies have demonstrated that an increased OLR can lead to substantial improvements in hydrogen production, highlighting its importance as a controllable variable.^56^ The integration of advanced modeling techniques, such as ANFIS contributes to the efficiency in predicting the operational parameters like transmembrane pressure, to improve reactor performance based on real-time data.^57^ Additionally, combining ANN with response surface methodology (RSM) has been successful in optimizing parameters such as substrate concentration, nutrient additives, and biomass dosage, leading to improvements in yield and minimizing inhibitory byproducts.^58^ These advanced, data-driven approaches streamline parameter prediction and operational adjustments, supporting greater efficiency and productivity in biohydrogen generation within batch reactor systems.

#### 3.1.3. Challenges related to batch reactor

Batch reactors face several challenges, especially in biohydrogen production through dark fermentation. One significant challenge is limited productivity, due to downtime during filling, and emptying phases, which reduces overall efficiency. Inconsistent product quality is another issue, with variations in operating conditions, substrate concentrations, and microbial activity causing fluctuations in each batch. Additionally, high substrate and by-product concentrations can inhibit microbial activity and hydrogen production, making it challenging to balance substrate levels for optimal productivity.^9^ The intermittent nature of batch reactors can lead to higher operational costs and lower economic viability compared to continuous systems, which can produce biohydrogen more consistently and efficiently. Furthermore, in order to avoid methanogenesis, the short HRTs, low pH, and high OLR are usually targeted.^52^ Addressing these challenges involves optimizing reactor design, improving operational protocols, and potentially integrating batch reactors with other systems to enhance overall productivity and efficiency.

### 3.2. Applications of CSTR

Hydrogen-producing microbes are thoroughly combined and suspended from the mixing pattern in the reactor liquid in a CSTR for continuous hydrogen production. Mass transport and a good substrate–microbe interaction can be achieved under such hydrodynamics. Short HRTs may result in biomass washout, which significantly limits the rates at which hydrogen can be produced.^59^ Microorganisms leveraging photosynthesis degrade organic compounds through photolysis to contribute to hydrogen production.^60^ Photosynthetic hydrogen generation can be achieved by microalgae and their different metabolic pathways using photosystems and light-independent fermentation. As a result of this process, a variety of small organic compounds are produced, including formate, acetate, and ethanol, in combination with hydrogen.^61^ Dark fermentation also produces low-weight organic compounds such as VFA and alcohols. This process of assimilation can be carried out by the genus of bacteria belonging to the genera *Prevotella*, *Lactobacillus*, *Clostridium*, *Selenomonas*, *Megasphaera*, and *Enterobacter*, with the most representative members belonging to these genera.^62^ CSTRs, being the most versatile reactor type, have been utilized by continuously introducing authentic wastewater to achieve high-rate BHP.^63^

#### 3.2.1. Influence of operating variables in CSTR

The performance of the reactor is dependent upon its mixing dynamics and structural configuration, as well as the speed of mixing, activation, and aggregation of inoculum which acts as the primary culture to instigate BHP. The continuous production of biohydrogen is predominantly achieved through the implementation of CSTRs, which is affected by various parameters as given in Table 2. In the process of using starch as a substrate in BHP, it was observed that butyrate to acetate ratio was a crucial factor affecting biofuel generation at long HRTs.^74^ When analysing the results of using pig slurry as a substrate instead of starch, it was observed that pig slurry can be used directly to create hydrogen continuously, and the combination of the high temperature (37 °C in the ambient range and 55 °C in the thermophilic range) and short HRT of around 24 h was sufficient to inhibit methanogenesis.^75^ Pre-treating the inoculum helped to enhance the activity of bacteria that produce hydrogen and reduce the activity of methanogens. Biohydrogen production from organic solid waste is affected by various factors like OLR and BHP rate and other particular operation conditions like the mixing and design of the reactor, mixing speed, activation, and accumulation of inoculum. Here, food waste can be utilized in batch and continuous processes as a substrate for dark fermentation to produce hydrogen.^76^ Waste bread has a high carbohydrate and protein content, due to which it is considered an appealing substrate for the synthesis of biohydrogen. Glycolysis is the final stage in biohydrogen generation when the sugars are hydrolysed into glucose and amino nitrogen for use followed by the utilization of nutrients by H_2_ producing microbes.^77^

**Table 2:** Application of CSTR reactors for BHP and their operating conditions

Substrate	Micro-organism	OLR	pH	Temperature	HRT	BHP rate	Reference
Poplar leaves	*Enterobacter aerogenes ATCC 13048*	200 g COD/L per day	5–8	30–40 °C	14 h	0.6 mol H_2_/mol per h	64
Date waste	*Thermotoga maritima*	204 g COD/L per day	7.0	80 °C	4 h	0.002 to 0.017 mol H_2_/L per h	65
Peanut shell	*Firmicutes*, *Bacteroidetes* and *Proteobacteria*	12 g COD/L per day	4.85–5	60 °C	6 h	0.58 L H_2_/L per day	66
Acidic cheese whey	*Clostridium* and *Lactobacillus* species	309 g COD/L per day	5	55 °C	4.5 h	3.2 L H_2_/L per day	67
Sugarcane biorefineries	*Clostridium* and *Thermoanaerobacterium*	30–120 g COD/L per day	5.0–6.0	55 °C	8–16 h	3.63 L H_2_/L per day	2
Pig manure	*Enterobacter aerogenes*	6.5 g COD/L per day	5.5	35 °C	3 days	15.8 L H_2_/L per day	68
Waste molasses	*Propionibacterium*, *Desulfobulbus*, *Methylobacterium*, *Clostridium*	70 g COD/L per day	5.7	60 °C	6 h	0.417 L H_2_/L per day	69
Sewage sludge and wine vinasse	*Eubacteria*	51.4 g COD/L per day	5.0–6.0	55 °C	12 h	35.19 L H_2_/L per day	70
Catering waste	*Methanosaeta concilii*	91.5 g COD/L per day	6.5	27 °C	24 h	3.84 L H_2_/L per day	71
Xylose	*Lactobacillus* and *Sporolactobacillus* species	21.61 g COD/L per day	7.5	30 °C	3 h	30.26 ± 1.19 L H_2_/L per day	72
Rice straw	*Ruminiclostridium* species	20 g COD/L per day	6.5	55 °C	3 days	24.8 L H_2_/L per day	73

In an effort to increase the volumetric hydrogen generation rate and the hydrogen yields, the impact of various HRT and OLRs with cheese lactoserum powder solution was examined. It exhibited the highest hydrogen molar yield of 2.8 mol H_2_ per mol lactose at an OLR of 138.6 g lactose/L per day and a HRT of 6 h. This combination resulted in the highest volumetric hydrogen production rate of 0.046 mol_H_2__ L^−1^ h^−1^. The microbial communities enriched at each culture condition were investigated using polymerase chain reaction – denaturing gradient gel electrophoresis, and the major metabolites produced were also observed. A clear dominance of *Clostridium* species, specifically *Clostridium butyricum* CM-C86 and *Clostridium butyricum* CM-C97 was detected.^78^ The microbial ecology of hydrogen-producing and lactate/acetate-utilizing bacteria that were enriched in the CSTR was examined using molecular techniques. The cloning and sequencing indicated that *Clostridium tyrobutyricum* was considered the major hydrogen-producing bacteria in the CSTR fed with lactate and acetate, which resulted in OLR of 55.64 g COD/L per day, for fermentation along with 6.60 mol H_2_ per mol lactose.^79^ A laboratory-scale CSTR employing hybrid immobilization with a substrate concentration of 60 g L^−1^ of galactose was operated at an OLR of 120 g/L per day and a HRT of 3 h. The reactor exhibited a peak BHP rate of 25.9 L H_2_/L per day, while a 6 h HRT yielded the maximum hydrogen yield of 2.21 mol H_2_ per mol galactose. Briefly, reducing the HRT to 2 h resulted in a recorded BHP rate of 39.65 L H_2_/L per day; however, the process failed to sustain due to the rapid washout of the hydrogen production consortium.^63^ The integration of a CSTR with an UASB reactor was explored in a two-stage process aimed at biohydrogen and methane production. Under optimal HRT of 3 h, the CSTR yielded methane at a rate of 2.25 L/L per day and achieved a maximum BHP rate of 17.5 L/L per day.^80^ Additionally, a separate investigation utilized a thermophilic mixed microbial culture as the inoculum, evaluating the fermentative hydrogen generation potential of waste and sludge obtained from a tofu-processing anaerobic digester, both in batch and continuous operation modes. At 4 h HRT, higher yields of 2.3 mol H_2_ per mol glucose equivalent and BHP rate of 12 L H_2_/L per day were attained.^81^

#### 3.2.2. Optimization strategies

The optimization in CSTR primarily involves adjusting key parameters like OLR, HRT, and leveraging granular sludge for higher hydrogen yields. Higher OLRs, when combined with a controlled biomass concentration, have been shown to enhance BHP by optimizing nutrient availability and substrate utilization, demonstrating the importance of tuning these parameters for maximum yield.^82^ Shortening the HRT has also proven beneficial, as it encourages granular sludge formation, improving biomass retention and overall reactor efficiency by maintaining a higher concentration of active biomass. Furthermore, Computational Fluid Dynamics (CFD) has been instrumental in optimizing mixing dynamics within CSTRs, particularly in horizontal CSTR configurations, where studies have demonstrated that fine-tuning the agitation speed enhances biohydrogen yield by promoting better substrate-contact and nutrient distribution.^83^ For more complex predictive control, machine learning models, such as backpropagation neural networks (BPNNs), have been applied to optimize BHP.^84^ These approaches reflect the significant role of parameter optimization, advanced simulation, and predictive modeling in enhancing biohydrogen production in CSTR systems.

#### 3.2.3. Challenges with CSTR

The performance of controlled BHP within a CSTR is contingent upon the simultaneous interaction of substrates and microorganisms, which occurs in an ongoing manner. A well-designed stirrer ensures adequate stirring and effective mass transfer while minimizing shear tension on biomass. However, because of short HRT and wash out, its performance has decreased.^12^ The main cause of this constraint is the poor settling characteristics of biomass. By physically retaining the microbial biocatalyst, this restriction can be removed. A metabolic shift toward solvent formation may also be the cause of poor hydrogen generation at greater OLR. Other major obstacles include the high operating costs and the economics of the BHP systems now in use. These challenges need to be addressed to ensure the sustainable and efficient BHP from industrial wastewater using CSTRs and other bioreactors.^85^ It has recently been suggested that immobilizing microorganisms on supportive materials or allowing hydrogen producers to self-granulate or flocculate could help retain germs, which in turn increases the output of biohydrogen.^86^ A range of physical or biological immobilization strategies can be used to improve the capacity of CSTR to produce hydrogen and retain biomass from the cells. Additionally, reducing operational costs through energy-efficient equipment and cost-effective materials can enhance the economic viability of BHP systems.

### 3.3. Applications of PBR for BHP

PBRs are widely used in BHP due to their versatile nature and efficiency. For continuous BHP, PBRs are suitable for certain biohydrogen-producing species such as *Clostridium*.^87^ To allow for the fast transfer of biohydrogen and to lessen the strong effect of partial pressure and supersaturation of biohydrogen, a horizontal fixed bed reactor was designed with pH stabilized at 5.9. At 6 h HRT and OLR of 0.88 g L^−1^, maximum affordable conditions for BHP by *Clostridium butyricum* in mixed culture was found. PBRs also have high biomass concentration as the immobilized microorganisms attached to the solid support material, provides a large surface area for the microorganisms to grow and multiply. These immobilized microorganisms are more stable and less prone to washout, therefore improving their ability to operate for longer periods of time.^13^ PBRs only need minimum amount of additional equipment and can be used in small commercial units. Other advantages of these reactors include easy extraction of catalyst from reactor effluent stream and the wide variety of times at which these reactors can be operated.

#### 3.3.1. Influence of operating variables in PBR

The composition and concentration of the substrate are critical in BHP. The substrate composition consists of various organic compounds such as simple sugars, complex carbohydrates, fats, and proteins present in the feedstock. This composition has the ability to influence the metabolic pathways of hydrogen-producing microorganisms by affecting the pH and VFA concentration.^88^ The higher substrate concentrations can lead to increased BHP, but excessive concentrations may inhibit substrates or lead to the accumulation of fermentation byproducts such as VFAs. On the other hand, lower substrate concentrations may reduce hydrogen yields by confining the growth and activity of microorganisms.^89^ The selection of specific microbial strain is important as different strains exhibit varying capabilities for BHP, substrate utilization, and tolerance within a packed bed reactor.^87^ Temperature is also a vital factor which affects the metabolic activity of microorganisms and the BHP rate. It was seen that in a certain range, temperature increment led to improvement of hydrogen producing bacteria during FHP, but it was also seen that at higher levels, this led to decrease in BHP.^90^ Thermophilic microorganisms have demonstrated higher BHP rate than mesophilic microorganisms, as they can produce hydrogen continuously at high rates, even at low HRT.^88^ The optimal temperature for fermentative BHP was found to be around 37 °C in mesophilic range and around 55 °C in thermophilic range and was not necessarily the same.^91^ Several studies were carried out with substrates such as sucrose and xylose in a PBR with municipal sewage sludge as inoculum.^92^ The optimal temperature was determined to be 40 °C for sucrose and 50 °C for xylose as substrates. Maximum hydrogen yield was studied and found to be higher for sucrose substrate (3.88 mol per mol sucrose) than xylose substrate (1.4 mol per mol xylose).

The pH is another critical factor that impacts the performance of PBRs for continuous BHP. During continuous BHP, the pH inside the reactor may drop due to organic acid accumulation, inhibiting metabolic activity and reducing the BHP rate.^88^ It was observed that increasing pH, led to increase of production ability in hydrogen producing bacteria. Optimal pH for fermentative BHP in a continuous PBR was reported by researchers to be 4.2 and 7 respectively.^93,94^ In another study, researchers investigated the BHP from traditional Chinese medicine wastewater as substrate in AnPBR by varying the pH range from 5.6 to 6.4 and also by trying different HRTs from 24 h to 6 h.^95^ HRT is also another factor that influences the BHP rate by affecting the residence time of microorganisms. The performance of an anaerobic packed bed reactor under different HRTs was studied from 6 h to 2 h to generate biohydrogen gas, with glucose as the substrate.^96^ HRTs varied between 2 h and 3 h, concluded that volumes ranged from 4.5 L per day and 5 L per day. The volumetric BHP rate saw an increase from 3.7 L per day to 4.73 L per day when HRT was changed from 2 h to 6 h, and at 6 h of HRT, the maximum yield of 0.89 mol was obtained for BHP. Interestingly, the hydrogen percentage remained stable at around 60% for all the HRTs examined. The BHP rate in a PBR by using paper mill effluent as a source of hydrogen was studied at HRT range of 2 to 24 h, and found that the highest BHP rate 6.21 L H_2_/L per day at the HRT of 2 h and the highest biohydrogen concentration of 41.5% in produced gas to be at HRT of 4 h.^14^ A longer HRT can also lead to the accumulation of organic acids, thereby reducing the BHP rate, whereas a shorter HRT reduces reactor volume and increases the BHP rate, resulting in a more compact and efficient system.^88^ An overview of the influence of various parameters on BHP is given in Table 3.

**Table 3:** Applications of PBR for BHP and their operating conditions

Substrate	Micro-organism	OLR	pH	Temperature	HRT	BHP rate	Reference
Pre-settled paper mill effluent	Anaerobic sludge	27.35 g COD/L per day	7	37 °C	2 h	6.21 L H_2_/L per day	14
Traditional Chinese medicine wastewater	*Clostridium butyricum*	328.2 g COD/L per day	6.6	37 °C	6 h	7.92 × 10^−3^ mol H_2_/L per h	95
Glucose	*Clostridium*, *Ethanoligenens*, *Lactobacillus*	40 g COD/L per day	5.7	35 °C	24 h	5.3 L H_2_/L per day	97
Glucose	*Clostridium*, *Ethanoligenens*, *Lactobacillus*	80 g COD/L per day	5.7	35 °C	24 h	4 L H_2_/L per day	97
Rice straw	Anaerobic seed sludge	—	5.3	37 °C	2 h	0.252 L H_2_/L per h	98
Winery wastewater	*Lactobacillus*, *pectinatus*, *Clostridium*	523 g COD/L per day	5.5	37 °C	5.5 h	1.44 L H_2_/L per day	35
Glycerol	*Rhodopseudomonas Palustris*	—	7	28 °C	—	0.012 L H_2_/g per h	99
Sugar beet molasses	*Clostridium pasteurianum*, *Clostridium tyrobutyricum*	850 g COD/L per day	4.4	—	8 h	208.3 L H_2_/L per day	100
Sugar beet molasses	*Clostridium pasteurianum*, *Clostridium tyrobutyricum*	790 g COD/L per day	4.6	—	9 h	100.1 L H_2_/L per day	100
Glucose	Anaerobic sludge	64 g glucose/L per day	5	48 °C	6 h	4.73 L H_2_/L per day	96

#### 3.3.2. Optimization strategies

In PBR, optimization relies on approaches such as statistical modeling, hybrid fermentation techniques, and innovative material utilization. RSM is commonly employed to mathematically model and analyze various process variables, identifying optimal production conditions that enhance BHP performance while minimizing experimental costs and effort. Through this approach, high-impact factors can be systematically adjusted to improve yields and achieve cost-effective production outcomes.^101^ Hybrid fermentation techniques, combining dark and photo-fermentation, present another promising optimization strategy in PBR systems. By coupling anaerobic carbohydrate fermentation with subsequent photo-fermentation stages, low molecular weight organic acids generated in the first stage can be efficiently converted into biohydrogen, leveraging the sequential transformation to increase hydrogen yield. Nanomaterials have also been recognized for their potential in optimizing yield in PBRs, as they enhance catalytic efficiency and provide stability during BHP. Studies indicate that nanomaterials can be integrated at various stages of the biohydrogen production process to boost reactivity and facilitate improved yield.^102^ This approach is associated with increased cost-efficiency and long-term viability for BHP technology, positioning nanomaterial-enhanced PBR systems as a competitive choice for sustainable hydrogen production.

#### 3.3.3. Challenges with PBR

The accumulation of fermentation byproducts or intermediate metabolites can possibly lead to substrate inhibition, which can affect the metabolic activity of hydrogen-producing microorganisms and decrease the BHP rates. Maintaining optimal temperature conditions is critical for maximizing BHP, but achieving precise temperature control, especially in large-scale reactors, can be challenging as the temperature gradients can lead to uneven microbial activity, impacting the overall hydrogen yields.^89^ Mass transfer limitations can also be a possibility, which can be overcome by using structured packing material with properties such as high surface area and porosity, so as to improve mass transfer and BHP.^103^ Reactor clogging is another common issue in PBRs when dealing with complex substrates or feedstocks with high solid content, which can impact the efficiency of BHP.^89^ The porosity of the packed bed material can contribute to channelling, where the liquid flows through the reactor without encountering the immobilized organisms, further reducing the efficiency of the process.^88^ Additionally, maintaining the operational stability of PBR is also a significant challenge. In the presence of fluctuating substrate compositions, environmental conditions, and microbial activities, PBRs may face issues with long-term operational stability, leading to inconsistent BHP rates and also poses challenges for the practical application of this technology.

### 3.4. Application of MBR for BHP

Membrane bioreactors (MBRs) are now emerging as one of the most efficient systems. The usage of MBRs has been concentrated on the BHP, mainly when combined with biological processes. MBRs can be classified mainly on their microbial structure and their performance. MBRs can be classified mainly on their microbial structure and their performance. Based on their microbial structure, MBRs are classified as aerobic (AeMBRs) and anaerobic MBRs (AnMBRs). AnMBRs are designed by adding together the membrane technology with traditional anaerobic fermenters. The main difference between these two types of MBRs is the lack of aeration system and low biomass production rate in AnMBRs. But in comparison to AeMBRs, AnMBRs only require lesser energy because of the lack of aeration system.^104^

#### 3.4.1. Influence of operating variables in MBR

BHP rates and yields in MBRs are influenced by several parameters, as shown in Table 4. Many studies have explored the different substrates suitable for BHP, including materials rich in carbohydrates such as simple sugars, starch, cellulose, and food related wastes.^112,113^ For the degradation of materials such as starch and cellulose, the time taken for fermentation is comparatively longer it needs to be converted into monosaccharides by hydrolyzation for BHP.^15^ Researchers found that the maximum yield of hydrogen, when using different carbohydrates as such from cellulose, starch and glucose, varied from 2.40 mol H_2_ per mol hexose^114^ to 3.33 mol H_2_ per mol hexose.^115,116^ Variations in HRT can impact microbial diversity in the system, with shorter HRTs leading to more efficient BHP by enriching biohydrogen-evolving bacteria while suppressing biohydrogen-consuming microbes. Optimal HRT ranges have been found to be between 0.5 to 12 h for substrates such as liquid waste streams in MBRs. SRT also plays a significant role in affecting BHP in MBRs, with optimal SRT varying depending on operational variables and different cases.^16^ Temperature is another critical factor in BHP, with studies showing that increasing the temperature in the mesophilic region improves BHP performance. However, temperature should be regulated to not exceed the mesophilic range, as this might affect BHP due to different properties of the microbial culture.^59^ Researchers have found that elevating the temperature helped the yield reach the highest levels of 0.36 mol per day at 30 °C to 34 °C, and the BHP rate of 1.42 mol H_2_ per mol glucose at 28 °C to 32 °C.^117^ pH plays an essential role in influencing the metabolic pathways and biohydrogen yield, with researchers finding the yield of BHP rate in the range of 5.2–6 when using bacteria of mixed cultures.^118^ Another set of researchers found that BHP rates from mixed sugars of glucose, xylose and arabinose to rice straw hydrolysate as substrates to be 28.52 L H_2_/L per day while studying the pH range from 5.5 to 6 at 3 h HRT.^104^ OLR is the quantity of organic material that is present per unit volume of reactor and finding the optimal OLR depends on parameters such as pH, type of substrate, concentration of substrate, sludge loading rate and the design of the reactor.^119^ Some studies found that sparging the system using nitrogen increased yield by 65%,^16^ and some other studies also found that BHP was amplified by 1.5 times when nitrogen sparging was done.^120^ Other than nitrogen sparging, there are other methods such as releasing gas continuously, stripping of vacuum and the availability of larger headspace to overcome the problem of high hydrogen partial pressure.

**Table 4:** Applications of MBR for BHP and their operating conditions

Substrate	Micro-organism	OLR	pH	Temperature	HRT	BHP rate	Reference
Diluted grape deposits	Dark fermentation of biomass	2 g COD/L per day	5	37 °C	8 h	4.5 L H_2_/L per day	105
Mixed sugar	*Clostridium pasteurianum*	2.54 g COD/L per day	5.5–6	—	3 h	28.52 L H_2_/L per day	106
Glucose (MBR with polyester mesh)	*Clostridium pasteurianum*	2.63 g COD/L per day	5.2	—	2 h	44.22 L H_2_/L per day	17
Glucose (MBR with stainless steel mesh)	*Clostridium puniceum*	2.99 g COD/L per day	5.2	—	2 h	51.64 L H_2_/L per day	17
Glucose	*Clostridium butyricum*	21.66 g COD/L per day	5.5–6	37 °C	2 h	50.37 L H_2_/L per day	107
Glucose	*Clostridium butyricum*, sludge originated biohydrogen producers	18.88 g COD/L per day	5.5–6	37 °C	2 h	58.57 L H_2_/L per day	107
Glucose	*Clostridium butyricum*	21.3 g COD/L per day	5.5–6	37 °C	2 h	58.86 L H_2_/L per day	108
Glucose	*Clostridium beijerinckii*, *Clostridium pasteurianum*, *Enterobactor* species	0.9 g glucose/L per day	4.8	37 °C	12.5 h	0.135 L H_2_/L per h	109
Food waste	*Clostridium* species	9.42 g COD/L per day	5.5–6	37 °C	8 h	7.09 L H_2_/L per day	110
Xylose	*Clostridium* species	17.37 g COD/L per day	—	37 °C	3 h	30.26 L H_2_/L per day	72
Algal biomass	*Clostridium* species, *Anaerostipes* species, *Caproiciproducens* species	21.4 g COD/L per day	5.5	—	3 h	21.58 L H_2_/L per day	111
Food waste	*Clostridium thermopalmarium*, *Clostridium butyricum*	18.61 g COD/L per day	7	55 °C	120 h	0.146 L H_2_ per g volatile solid added	112

#### 3.4.2. Optimization strategies

Optimizing BHP through MBRs can be effectively achieved by integrating MBRs with Microbial Electrolysis Cells (MECs). This combination enhances BHP by applying an external voltage to drive hydrogen generation, where organic compounds are biologically catalyzed into hydrogen, thus improving the reactor's overall efficiency and yield.^121^ Additionally, real-time monitoring systems play a critical role in MBR optimization by continuously tracking parameters such as BHP rate, pH, and temperature. Real-time data enables precise adjustments that respond to fluctuations in reactor conditions, promoting steady BHP output and efficiency. Comparative studies between dynamic MBRs and conventional MBRs demonstrate the advantages of dynamic MBRs, which offer greater cost-effectiveness, energy efficiency, and enhanced filtration flux.^111^ These performance benefits make dynamic MBRs a promising alternative for scalable and efficient biohydrogen production systems.

#### 3.4.3. Challenges with MBR

Even though BHP through MBRs is a well-established and mature technology, there are is still a need to overcome barriers to practical applications such as low yields and low BHP rates. Low yield and BHP rates are the two most frequent challenges in MBRs. More research is needed to reduce the technical challenges, such as high cost of BHP, storage, and delivery of biohydrogen.^59^ The main challenges faced by MBRs include its need for high-frequency cleaning, membrane fouling,^122^ increment in concentrations of methane in effluent,^123^ and need of post treatment for the recovery of nutrients.^124^ Membrane fouling is another major challenge for these reactors as it reduces the permeability of the membrane, therefore affecting the efficiency of BHP. To control membrane fouling, operating conditions such as temperature, pH, HRT, SRT and membrane characteristics should be regulated.^104^ An effective way to overcome this membrane fouling is to treat the membrane with chemicals, but this can result in shorter membrane lifetime.^16^ The limit to which the microbial community of BHP can extend to is yet to be found. The undiscovered biological diversity may have the potential to set up new mechanisms for integrating and utilizing microbial resources.

## 4. AI integration

**Fig. 3:**
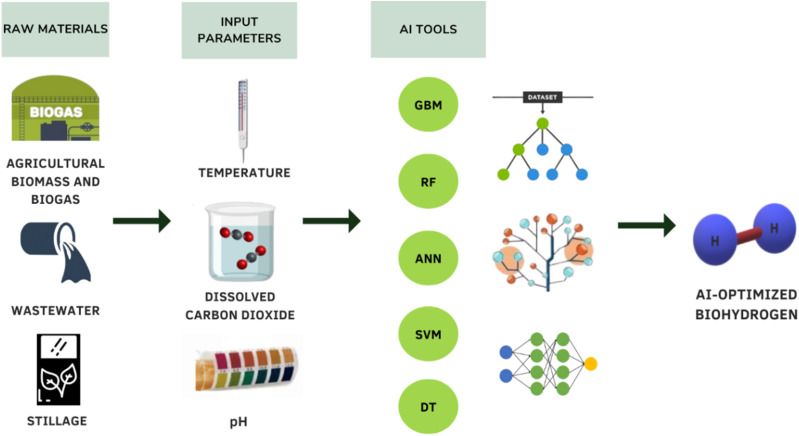
Applications of AI in BHP.

The BHP from dark fermentation is a highly complex procedure, but this process can be made efficient and economical by employing various AI techniques. Recent research has shown that producing biohydrogen from agricultural biomass is a comparatively green technique because it requires lower temperatures and pressures during the synthesis process. Due to its economic and environmental potential, BHP from agricultural waste has attracted attention on a global scale. Design and optimization techniques can prove to be useful to scale-up bioprocess technology to the industrial level. Innovation is supported by ML in a number of domains, such as waste-to-energy network design and online process control. The manufacturing processes can be improved and modelled to reach a maximum yield and BHP rate, as shown in Fig. 3. The main objective of modelling is to improve yields by optimizing the processes involved in producing these biofuels.^125,126^

ML tools are increasingly integrated into BHP processes, offering notable benefits in efficiency, reliability, and cost reduction. By utilizing ML algorithms to analyze data patterns in BHP, these tools allow for the identification of critical trends, aiding in performance optimization. ML, as a reliable predictive model, assists in estimating process efficiency and predicting yield outcomes across BHP stages, making it a valuable tool for enhancing overall productivity while lowering operational expenses.^127^ Since ML models rely heavily on pre-existing data, they provide rapid response capabilities by approximating complex, non-linear behaviors of input parameters in BHP, thereby allowing for real-time monitoring and control.^128^ This adaptability is especially advantageous in BHP due to the variable nature of input conditions, enabling quick, data-driven adjustments to optimize yield and maintain process stability. By offering insights into parameter interactions and predicting outcomes, AI-driven models play a critical role in efficiently managing BHP processes with minimal trial-and-error experimentation.

### 4.1. Artificial neural networks for BHP

The production of biohydrogen involves several complex steps, and the application of ANN to different steps can result in enhanced outputs and effective production. Three ANN models used to predict parameters such as BHP rate, accumulated hydrogen production and hydrogen yield, by determining the profiles of volatile fatty acids generated during manufacturing using a developed ANN model reported highly accurate results (*R*^2^ > 0.987).^129^ ANN has been used to estimate BHP rate in a CSTR, with a minimum training error of 0.0438 and maximum *R*^2^ value of 0.92, by using input parameters such as HRT, biomass concentrations and pH to model a separate neural network.^130^ An ANN model with a feed-forward algorithm, four input and two hidden layers was used to model BHP from a continuous anaerobic sludge blanket filter using 109 data points for training. The ANN model correlated the inputs and outputs using optimum weights with *R*^2^ value of 0.9981.^131^ ANN modelling carried out on the efficiency of BHP on sugar cane molasses revealed a *R*^2^ value of 0.91, revealing that ANN has great accuracy in modelling relationships between input and output parameters for fermentative BHP. In this study, back propagation algorithm was used to train the ANN model.^132^ The estimated BHP in fermentative processes and the quantity of biohydrogen released from genetically-altered *E coli* bacteria was predicted using BPNN with twelve nodes and one hidden layer. The study reported a correlation coefficient (*R*^2^) of 0.955.^126^ Similarly, back propagation algorithm modelled to train and estimate error, in a process meant to predict hydrogen yield during the dark fermentation of coffee mucilage wastes give an error less than 0.002.^133^ Another back propagation model of BHP in a hybrid up-flow anaerobic sludge blanket reactor made use of four inputs and one output reported training regression coefficient *R*^2^ of 0.929 in the operation phase of BHP.^134^

ANN have been effectively integrated into BHP, especially in optimizing complex bioprocesses with multiple interdependent steps. By modeling intricate interactions between input and output parameters, ANN enables a better understanding of how different inputs affect the biohydrogen yield without requiring a comprehensive mechanistic understanding of the underlying processes.^135^ In scenarios where governing frameworks are either undefined or complex, ANN provides a robust solution by accurately representing these non-linear relationships, facilitating real-time decision-making.^136,137^ ANN proves particularly useful in tasks such as prediction, monitoring, and process control in BHP, which are critical to optimizing production costs and time efficiency. It enhances the process by forecasting performance trends, identifying optimal operating conditions, and adjusting parameters for consistent yield maximization.^56^ By reducing reliance on extensive experimental setups and time-consuming manual adjustments, ANN contributes to cost-effective, reliable BHP operations, underscoring its value as a primary AI tool for improving BHP productivity.

### 4.2. Support vector machine for BHP

SVM is another very important algorithm used majorly in classification and troubleshooting. BHP from wastewater using the dark fermentation procedure was predicted using different ML models including SVMs. The SVM model resulted in a high determination coefficient (*R*^2^) of 0.885, which was dependent on a variety of parameters such as environmental temperature, pH of solution, materials used and HRT.^138^ Another investigation reported that SVM had an improved performance in terms of classification and regression analysis when compared with other traditional statistical algorithms.^128^ SVM used to model the process involving hydrogen generation from organic wastes reported a high *R*^2^ of 0.988 of the hydrogen yield.^139^ Different ML models were used to check the yield of hydrogen produced from supercritical water gasification of biomass, which resulted in an *R*^2^ value of 0.9768 and a root mean square error of 0.8740 for the SVM model, which performed better than their ANN model.^140^ SVM has been reported to be highly affected by noisy inputs, has high computation cost and is a slower, more expensive method than ANN.^128^ However, it is a huge asset when users need to control the output error, and in cases where input data is highly disorganized.^141^ One major advantage of using SVM algorithm is that it can allow users to specify or set the threshold levels for the amount of error that should be endured by the model.^142^ This provides greater control over the error and leads to better, more accurate results.

### 4.3. Other algorithms for BHP

Decision Tree (DT) is a widely-used ML algorithm used for creating classification systems relying on several variables. Random Forest (RF) is an algorithm that prevents data points on different decision trees from converging by using bootstrap aggregation.^143^ In a study of microalgal hydrogen production, the researchers noted that RF was resilient to over-fitting, a situation where the learning fits completely over the data used to train it. This affects the ability of model to foresee future outcomes.^141^ Gradient boosting machine (GBM) is an improvement model used along with regression trees to generate strong, highly reliable, and comprehensible algorithms. In comparison to other ML and regression models, GBM is more flexible, has superior precision and involves less usage of processing power.^144^ Researchers reported an *R*^2^ value of 0.976 in the validation phase and 0.805 in the testing phase with RF. Furthermore, the mean squared error was only 0.004 in the validation phase and 0.023 in the testing phase.^138^ In case of GBM, the *R*^2^ and mean squared error value of were 0.985 and 0.002, respectively in the training or validation phase and 0.802 and 0.023, respectively in the testing phase. All models showcased similar strengths (*R*^2^ value), and almost identical mean squared error values. However, while comparing mean absolute error, SVM to show least error, followed by GBM and RF. Another gradient boosting DT to ascertain properties of cellulose-rich materials produced from lignocellulosic biomass reported a low *R*^2^ value of approximately 0.90.^145^ In situations where only limited or partial inputs are present, RF holds a clear advantage over other ML algorithms like ANN.

## 5. Life cycle and technoeconomic analysis

**Fig. 4:**
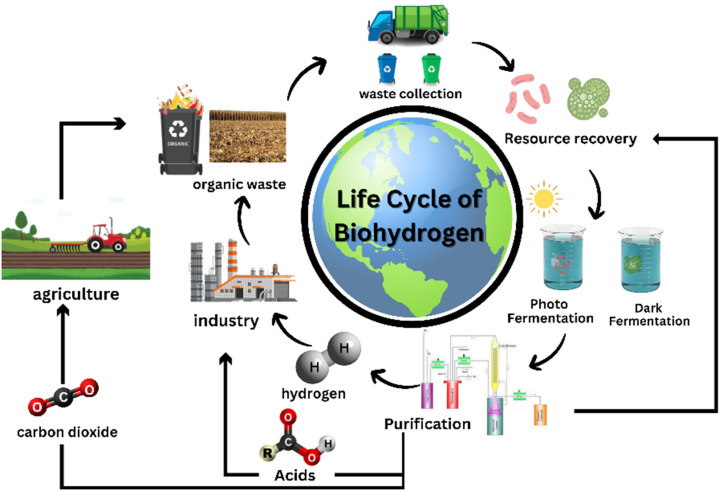
Life cycle of biohydrogen.

LCA is considered a crucial requirement to prove the sustainability of the process or technology.^146^ Though not sustainable, fossil fuels are widely used to produce hydrogen as the process involved is inexpensive as compared to greener alternatives.^113^ BHP from organic sources helps in pollution control as well as gives us an alternative source of energy, as shown in Fig. 4. The researchers observed that around 75% of the total greenhouse gas emissions could be attributed to the supply of heat and more than 15% was due to electricity used in the entire process of biohydrogen produced through dark photosynthesis.^147^ Another LCA study revealed that among various BHP processes like gasification of softwood and steam reforming, production of biohydrogen through eucalyptus leaves and their gasification is responsible for the least carbon footprint of approximately 1.6 kg of CO_2_ equivalent per kg hydrogen.^148^ The two processes extensively used to produce hydrogen from ethanol, *i.e.*, dehydrogenation and steam reforming showed that using the steam reforming method released carbon even though it was cheaper, whereas dehydrogenation had zero carbon emission coupled with the release of industry-relevant chemicals like ethyl acetate and acetaldehyde.^149^ Three approaches have been listed from a life-cycle viewpoint for evaluating the methods to valorize stillage produced during bioethanol production *via* composting, one-stage anaerobic digestion and dark fermentation followed by anaerobic digestion.^150^ Although anaerobic digestion was a more favourable approach, there was not much difference between the LCA of dark fermentation and anaerobic digestion for anyone to make a reasonable distinction between them.

Technoeconomic analysis are conducted to verify the economic feasibility and establish the optimisation pathways. Though costs to produce biohydrogen may differ depending on the procedure used or method followed, it takes an estimated $3.2–$48.96 to produce one kg biohydrogen through dark fermentation. Pretreatment costs vary from procedure to procedure and can sometimes contribute to more than 50% of the total operational cost.^151^ Raw materials used to maintain the BHP plants may contribute up to 80% of production costs. An investigation carried out to compare the cost of hydrogen production from conventional sources and fermentation concluded that producing hydrogen through the former using methods such as electrolysis, natural gas reforming and coal gasification is less expensive.^113^ A comprehensive breakdown of expenditure related to BHP through dark photosynthesis found the plant costs contributed to 28% while direct and indirect costs contributed to 27% of the total capital expenditure. Operational expenditures, on the other hand, are expenses made to keep the production plant up and running. These include resources like heat, water and electricity.^147^ Another study on TEA listed the minimum costs required to produce biohydrogen through several methods and claimed that at $1.20 per kg hydrogen, gasification is the most feasible process, followed by anaerobic digestion at $1.25 per kg. However, hydrogen generated from domestic biomass was less expensive ($2.40 per kg hydrogen) than that produced from agricultural residue ($4.55 per kg hydrogen).^148^ An all-inclusive analysis of the costs associated with different thermochemical technologies to produce hydrogen were found to be economically feasible with an approximate payback time of 5.10 to 7.18 years.^152^ Some improved technologies like photo-bioreactors and solar photovoltaic-based production plants can produce biohydrogen at much cheaper rates of $2 per kg and $14 per kg, respectively.^153^

## 6. Future directions

Research efforts are increasingly focused on biohydrogen production as a viable and efficient alternative energy source, driven by the escalating decline of fossil fuels and its consequential effect on the natural ecosystem. Currently, steam reforming of methane is the most cost-effective technique of hydrogen synthesis, contributing more than 50% of world's hydrogen generation.^154^ The focused problem is costing, which is higher because of reduced hydrogen yields and the process instability. Fermentation process provide lower yields with low conversion efficacy, which may be related to the biomass structure, necessitating sophisticated processing techniques during the process.^155^ In addition, the hunt for cheaper biomass is critical. Operating time, capital investment, electricity produced, and conversion efficiency are all factors that influence biohydrogen production rate during the electrolysis process.^156^ A rising strategy for dealing with the main economic concern is the shift toward integrated hydrogen production systems capable of increasing hydrogen yields. In addition, combined dark and photo fermentative procedures (two-stage integration) are being used to assist hydrogen generation techniques.^157^ The incorporation of hydrogen with the biodiesel manufacturing process can boost energy recovery by 90.3% over single stage hydrogen synthesis, which has to be thoroughly investigated.^158^ A hydrogen molar production of 77% of the theoretical maximum of burned feedstock was achieved during 15 days of operation using integrated dark-photo-fermentative technique.^159^ Furthermore, the use of synthetic enzymatic pathways based on a set of recombinant enzymes allows for virtually full conversion of various types of carbohydrates into ‘green’ hydrogen with stoichiometric conversion yields close to theoretical values. Although conversion rates and total prices need to be reduced, cost projections predict that in the future, this sustainable and ecologically friendly strategy will be comparable with thermochemically generated hydrogen.^160^ The extent of hydrogen production in the next years will be determined not only by scientific breakthroughs (improvements in efficiency through genetically modified microbes, bioreactor development, efficient manufacture of thermostable enzymes, among other things), but also by economic factors.

## 7. Conclusion

In light of the urgent need for sustainable energy solutions to address depleting fossil fuels and environmental concerns, biohydrogen production offers significant potential as a renewable energy source. This review highlights that optimizing reactor configurations and operational parameters, such as substrate concentration, microbial strain selection, temperature, pH, and HRT, is crucial for maximizing efficiency. However, the reactors face challenges like substrate inhibition, operational stability, and membrane fouling, which necessitate ongoing research and innovation to overcome these limitations. Key findings indicate that while each BHP method and reactor type presents unique advantages and challenges, optimizing operational parameters such as substrate selection, temperature control, and pH balance is critical for maximizing hydrogen yield and process efficiency. The integration of AI-driven tools into BHP processes represents a promising development, enabling predictive modeling, process optimization, and improved real-time control that could drive significant gains in efficiency and scalability. Furthermore, it is important for manufacturers to conduct LCA and TEA, which allows to decrease greenhouse emissions by using alternative production processes and minimizing costs by reducing direct and indirect plant costs.

Moving forward, future research should focus on overcoming these limitations through innovative reactor designs, AI integration, and sustainable waste management practices. As BHP technology advances, ongoing policy support, combined with rigorous research and development, will be essential to realizing the potential of biohydrogen as a foundation of the global renewable energy transition.

## Data availability

No primary research results, software or code have been included and no new data were generated or analysed as part of this review.

## Conflicts of interest

The author declared no potential conflicts of interest with respect to the research, authorship, and/or publication of this article.
